# MRI of trunk muscles and motor and respiratory function in patients with myotonic dystrophy type 1

**DOI:** 10.1186/s12883-019-1357-8

**Published:** 2019-06-19

**Authors:** Gro Solbakken, Bård Bjørnarå, Eva Kirkhus, Bac Nguyen, Gunnar Hansen, Jan C. Frich, Kristin Ørstavik

**Affiliations:** 1 Department of Neurology, Rheumatology and Rehabilitation, Drammen Hospital Vestre Viken Hospital Trust, P.O. Box 800, 3004 Drammen, Norway; 2 Institute of Clinical Medicine University of Oslo, P.O. Box 1171 Blindern, 0318 Oslo, Norway; 3 Department of Diagnostic Imaging, Drammen Hospital, Vestre Viken Hospital Trust, P.O. Box 800, 3004 Drammen, Norway; 4 Division of Radiology and Nuclear Medicine, Oslo University Hospital, Rikshospitalet, Oslo. P.O. Box 4950 Nydalen, N-0424 Oslo, Norway; 5 Faculty of Medicine, University of Oslo, P.O. Box 1130 Blindern, 0318 Oslo, Oslo Norway; 6 Department of Neurology, Section for Rare Neuromuscular Disorders, Oslo University Hospital, Oslo. P.O. Box 4950 Nydalen, N-0424 Oslo, Norway

**Keywords:** Myotonic dystrophy type 1, MRI, Muscle-size, Fat-infiltration, Trunk-muscles, Respiration, Mobility

## Abstract

**Background:**

Myotonic Dystrophy 1 (DM1) causes progressive myopathy of extremity muscles. DM1 may also affect muscles of the trunk. The aim of this study was to investigate fat infiltration and muscle size in trunk muscles in DM1 patients, and in an age and gender matched control group. Further, explore how fat infiltration and degree of atrophy in these muscles are associated with motor and respiratory function in DM1 patients.

**Method:**

We measured fat infiltration and trunk muscle size by MRI in 20 patients with genetically confirmed classic form of DM1, and compared these cases with 20 healthy, age and gender matched controls. In the DM1 group, we investigated correlations between MRI findings and clinical measures of muscle strength, mobility and respiration. We used sum scores for fat infiltration and muscle size in trunk flexors and trunk extensors in the analysis of group differences and correlations.

**Results:**

Significant differences between cases and controls were present for fat infiltration in trunk flexors (*p* = 0.001) and trunk extensors (p = < 0.001), and for muscle size in trunk flexors (*p* = 0.002) and trunk extensors (*p* = 0.030). Fat infiltration in trunk flexors were significant correlated to back extension strength (*rho* = − 0.523 *p* = 0.018), while muscle size in trunk flexors was significantly correlated to trunk flexion strength *(rho =* 0.506 *p =* 0.023*).* Fat infiltration in trunk flexors was significantly correlated with lower general mobility (*rho =* − 0.628, *p =* 0.003), reduced balance (*rho =* 0.630, *p <* 0.003) and forced vital capacity (*rho* − 0.487 *p* = 0.040).

**Conclusions:**

Trunk muscles in DM1 patients had significant higher levels of fat infiltration and reduced muscle size compared to age and gender matched controls. In DM1 patients, fat infiltration was associated with reduced muscle strength, mobility, balance and lung function, while muscle size was associated with reduced muscle strength and lung function. These findings are of importance for clinical management of the disease and could be useful additional outcome measures in future intervention studies.

**Electronic supplementary material:**

The online version of this article (10.1186/s12883-019-1357-8) contains supplementary material, which is available to authorized users.

## Background

Myotonic Dystrophy 1 (DM1) is a progressive autosomal dominant inherited multisystem disorder caused by a CTG nucleotide repeat expansion in the myotonic dystrophy protein kinase (DMPK) gene on chromosome 19. DM1 is one of the most prevalent neuromuscular disorders affecting about one in 8000 [1–4]. The motor impairments in DM1 are assumed to progress from distal to proximal in the extremities. Early involvement of the face and anterior neck muscles are common [2, 5, 6]. Spine deformity and general weakness are anecdotally reported to occur late in DM1 progression [4].

We recently documented early and severe impairment in trunk muscles when measured with manual muscle strength tests (MMT) [7]. The trunk impairment was correlated to lower general mobility, reduced balance and use of Bilevel Positive Airway Pressure (BiPAP). The severity of the impairments and their correlation to the size of the CTG expansion suggested that DM1 could cause myopathy in trunk muscles [7]. There is a need for more knowledge about trunk muscle impairment in DM1 and how trunk muscle weakening may influence function [8]. Falls are prevalent in DM1 patients, and knowledge of whether myopathy in trunk muscles are related to mobility or balance, is of clinical importance [9]. Respiratory function may also be impaired [3, 10], and represents the main cause of mortality in the group [11]. The abdominal muscles are important for respiratory function [12] and involvement of trunk muscles may therefore indicate a need for ventilatory support [7].

The MMT, though easy to perform in the clinic, does not specify the cause of the weakness. Muscle biopsies in DM1 patients have shown fat infiltration and type 1 fiber atrophy in affected extremity muscles [2]. MRI has turned out to be a useful non-invasive measure of myopathy in muscular dystrophies [13–15]. The few MRI studies of trunk muscles in DM1 have focused on fat infiltration only [13, 14, 16]. The need for atrophy measures in future MRI studies of muscular dystrophies was recently addressed in a review of muscle MRIs [6]. Muscle size measured by MRI is highly related to strength measured by MMT and quantitative handheld dynamometers [17]. Significant correlation between strength and muscle size has also been found in an animal model of DM1 [18]. Edema is previously documented in a large number of DM1 patients in the extremity muscles as well as the erector spinae [13, 14]. Further, edema is found both in muscles with and without fat infiltration or atrophy, and is suggested to precede fat infiltration [14]. To describe muscle involvement in trunk muscles in DM1, we investigated edema as well as fat infiltration and atrophy.

Fat infiltration of muscles has been studied in healthy subjects and is related to age, gender and BMI [19, 20]. These variables are therefore to consider when interpreting MRI findings in DM1 patients. No previous MRI study of trunk muscles in DM1 has compared patients with healthy controls. The aim of this study was to investigate muscle size (diameter and area) and fat infiltration in trunk muscles in DM1 patients, and compare the results to an age and gender matched control group. In the DM1 patients we also aimed to explore whether the amount of fat and the size of the trunk muscles correlate to trunk muscle strength, respiratory function and other motor measurements.

## Methods

### Recruitment and inclusion

This study is part of a larger observational study on patients with DM1 classic form (defined as disease onset after 10 years): *“Myotonic Dystrophy type 1. Mechanisms, course of progression and optimization of development”*, focusing on CTG repeats, somatic and cognitive symptoms and pain. The first publication was published in 2016 [7]. A total of 50 DM1 patients have been recruited from different parts of Norway. In the present MRI study, patients living in the region where MRI was performed and without contraindications such as pacemaker or other metal implants, were included. In total, 22 patients were invited and from September 2016 until June 2017, 20 patients who consented to participate in the MRI study were included. 20 healthy age and gender-matched controls were recruited through written announcements to employees at the hospital departments. The invitation to participate as a control was not restricted to employees. Participants were included consecutively, there were no drop-outs after consent.

### Disease duration and CTG

In patients, disease duration was calculated based on time between onset of typical DM1 symptoms such as myotonia, loss of strength, cataract or arrhythmia and the date of the MRI investigation. Southern blot analysis [1] for number of CTG repeats was obtained from all patients within three years from the time of the MRI procedure.

### Evaluation of motor function

#### Skeletal muscle strength

MMT was used for assessing strength in the trunk flexors; measured by the curl up, and strength in the trunk extensors; measured by trunk extension from prone (see Additional file 1: Table S2 for procedures) in both controls and DM1 patients at the time of the MRI [21]. Muscle strength was scored with an adapted 0–3 Medical Research Council (MRC) scoring, from MRC 0–5, see Table 1. The ordinary MRC 0–5 scale (Additional file 1: Table S2) has been criticized for its unequal categorical width, providing only ordinal data, and for low discrimination between categories when used in clinical practice [22–24]. To counteract these limitations, the MRC 0–5 scale was recoded to a modified 0–3 scale (Table 1), according to Vanhoutte et al. [24]. By Rasch modelling of strength measure data from 1065 patients with different neuromuscular disorders (including DM1), Vanhoutte et al. documented reliability and restored thresholds enabling the modified 0–3 scale to be analyzed as an interval scale [24].

**Table 1:** Medical research council MMT; recoding from six-point to modified 0–3 scale

Six-point ordinal scale	0–3 Interval scale
0 = No muscle contraction	0 = Paralysis
1 = Flicker or trace of muscle contraction	1 = Severe weakness defined as > 50% loss of strength
2 = Active movement with gravity eliminated	
3 = Reduced power but active movement against gravity	2 = Slight weakness < 50% loss of strength
4 = Reduced power but active movement against gravity and resistance	
5 = Normal power against full resistance	3 = Normal strength

### Evaluation of function in the DM1 group only

Rivermead Mobility Index (RMI) is a questionnaire that measures general mobility. RMI consists of 15 items. The sum range is 0–15. A high sum indicates better mobility performance. RMI is found reliable and valid and recommended for DM1 patients [25, 26] and was measured in the patient group only.

Timed Up & Go (TUG) expressed in seconds, is used for evaluation of mobility and balance. The time used to rise up from a chair with armrest (0.45 m high), walk three meters, turn, walk back and sit down, was recorded. The patients were instructed to walk in a safe manner, and as fast as possible. The procedure was done twice, and the second test was recorded. Acceptable test-retest stability has been documented in DM1 patients [27, 28]. TUG was only measured in the patient group.

Walking capacity, expressed in meters, was measured by using the Six Minute Walk Test (6MWT) according to the American Thoracic Society (ATS) guidelines on a track in a corridor [29], one exception being the track distance, which in this trial was 20 instead of 30 m. The 6MWT is proven to be feasible and reliable as a measure for walking capacity in DM1 [30] and was measured only in the patient group.

Forced Vital Capacity (FVC) [31], expressed in % of predicted values was recorded from chart information for the patients who had completed this examination of respiratory function as part of their follow up programs in the local hospitals.

### Other measurements

Body Mass Index (BMI) [32] expressed in kg/m^2^ was calculated for all persons included, both cases and controls.

### MRI measurements, interpretation and scoring

MRI was completed in 20 DM1 patients and 20 controls. MR imaging was performed using 1.5 T. MR unit (Magnetom Avanto, Siemens, Erlangen, Germany) with phased array body coils. The trunk muscles, from the 11th thoracic vertebra level to the level of the lesser trochanter of the hip, were examined by 1): a transversal T1-weighted turbo spin echo sequence performed in two steps with breath holding (TE/TR 9/350 ms; echo train length 3; slice thickness 5 mm; distance factor 200%; field of view (FOV) 370 mm; matrix size 320 × 320; in-plane resolution 1.2mm^2^; number of slices 15; parallel imaging GRAPPA 2, 2): a transversal T2 turbo inversion recovery magnitude (TIRM) (TE/TR 31/ 3700 ms; TI 180 ms; echo train length 8; slice thickness 5 mm; distance factor 200%; FOV 370; matrix size 288 × 320; in-plane resolution 1.2mm^2^; number of slices was 30; GRAPPA 3 and 3): a sagittal T1-weighted turbo spin echo covering the columna (TE/TR 8.3/471 ms; echo train length 3; slice thickness 4 mm; distance factor 20%; FOV 400 mm; matrix size 256 × 320; in-plane resolution 1.3mm^2^; number of slices 15; GRAPPA 2. Total scanning time was approximately 15 min.

The MRI scans were scored independently by two radiologists, with 20 years of experience within the musculoskeletal field. They were aware of age and gender but blinded for clinical information. The images were anonymized, and scored randomly, the readers were blinded for each other’s scorings and whether the images belonged to patients or controls. The rectus abdominis -, the abdominal oblique -, the abdominal transverse -, the erector spinae -, the psoas - and the gluteus maximus muscles were scored. Muscle fat infiltration and muscle size were assessed on transversal T1-weighted images at preselected standardized levels that were identified with the help of the sagittal T1 weighted images. Fat-infiltration was scored according to the Mercuri-score, developed for use in muscular dystrophy, and widely used in reporting muscle fat-infiltration in myopathic muscles [33, 34]. The global degree of muscle fat infiltration (grade 0: no fat-infiltration; grade 1: fatty streaks; grade 2: less than 30% fat infiltration; grade 3: more than 30% but less than 60% fat infiltration; grade 4: more than 60% fat infiltration; grade 5: totally replaced by fat) was scored [35]. Grade 0 and 1 were defined as normal. The rectus abdominis muscles were scored in two intervals, above and below umbilicus. The erector spinae muscles were scored in three intervals: (above the L2/L3 disc level; between the L2/L3 and the L4/L5 disc level; and below the L4/L5 disc level.

The maximal thicknesses (mm) of the rectus abdominis muscles, the external and the internal abdominal oblique muscles and the abdominal transverse muscles were registered. The muscles were scored in a level above the umbilicus. The maximal thicknesses of the rectus abdominis muscles were also registered in a level below umbilicus.

The areas (mm2) of the erector spinae muscles were registered in the L1/L2, L3/L4 and L5/S1 disc levels [36]. The areas (mm^2^) of the psoas muscles were registered in the L4/L5 disc level. The areas of the gluteus maximus muscles were not analyzed.

All muscle sizes were assessed including the areas with fatty degeneration.

Muscle edema, interpreted when there were hyperintensity on TIRM images, was assessed at preselected standardized levels that were identified with the help of the sagittal T1 weighted images.

**Fig. 1:**
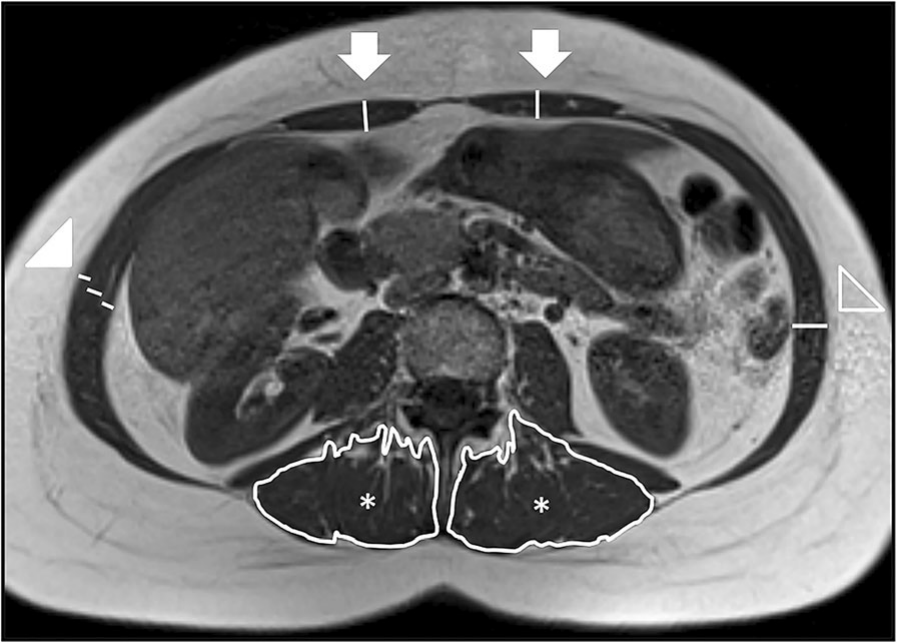
Transversal T1 weighted images in L3/L4 level in a female DM 1 patient illustrates the measuring of the thickness of the cranial rectus abdominis muscle (arrow), the three layers in the lateral abdominal wall; the external, internal and transversal abdominal (arrow head), and the sum of the three layers (open arrow head), and the area of the erector spinae muscle (*)

All measurements were done manually (Fig. 1).

### Statistical analysis

The SPSS 24 (IBM Corporation Armonk, NY, USA) was used for calculations.

Normal distributed variables were presented with mean, standard deviation (SD) and range. Non-normal distributed variables were presented with median and range. Assessment of group difference between cases and controls, and between patients with and without edema, was done by Independent sample T-tests and Man-Whitney U test when appropriate. Effect sizes (Cohens *d*) were calculated using the online social science statistics service: http://www.socscistatistics.com/effectsize/Default3.aspx. Cohens *d* at 0.2 were interpreted as small, 0.5 as medium and > 0.8 as large. Correlations between MRI measures and clinical measures were calculated by Pearson’s r and Spearman’s rho when appropriate. Correlations between MRI results and function are performed with sum scores for fat infiltration, and muscle size: 1) sum fat infiltration in trunk flexors (both the abdominal recti, the abdominal obliques and the abdominal transversus), 2) sum fat infiltration in trunk extensors (erector spinae L1/L2, L3/L4 and L5/S1), 3) sum muscle size in trunk flexors (both the abdominal recti, the abdominal obliques and the abdominal transversus), 4) sum muscle size in trunk extensors (erector Spinae L1/L2, L3/L4 and L5/S1).

A linear regression model with forced entry method was constructed to explore variables related to pulmonary function. Assumptions for Multiple linear regression was met: Linear relationship between outcome and independent variables, multivariate normality, homoscedasticity and no multi collinearity. *P*-values were set at two-tailed < 0.05, and exact values are reported when > 0.001. Bonferroni correction is used for adjustment of several statistical group comparisons. Based on power from previous MRI studies we calculated 40 participants to be sufficient (95% power) for answering the case control question. Interrater variability between the two radiologists reading the MRIs was calculated by interclass correlation (ICC (3.1) two - way mixed, consistency)). Mean ICC was 0.90 (excellent). In light of the high ICC only measures from one of the radiologists are presented in this study. Paired sample t-tests were used for comparison of left vs right side muscle size; Wilcoxon signed-rank test were used for comparison of left vs right side degree of fat infiltration.

## Results

### Group characteristics

There were no significant differences in age, gender and BMI between the patients and control group. The DM1 patients had decreased trunk extension and trunk flexion strength compared to healthy controls (Table 2). For information about muscle strength in the extremities and neck in the DM1 group, see Additional file 1: Table S1.

**Table 2:** Comparisons of characteristics of 20 DM1 patients and 20 controls

Characteristics	DM1 patients		Controls		Difference		
	Value	Range	Value	Range	*t*	p-level	Cohens-d
Men/women % 40/60	8/12		8/12				
Age years mean ± SD	39 ± 12.8	19–62	39 ± 12.8	16–61	0.087	0.931	0
BMI kg/m^2^ mean ± SD	25 ± 4.7	15–33	25 ± 4.5	18–34	0.113	0.911	0
*Strength trunk-flexion median	1.5	1–3	3	3–3		< 0.001	
*Strength trunk-extension median	2	1–3	3	2–3		< 0.001	

*Muscle strength is measured with the MMT, using the adapted 0–3 MRC score

Descriptions for the function measures TUG, 6MWT, RMI and FVC as well as the DM1 characteristics CTG size and disease duration are shown in Table 3. None of the patients were treated with Mexiletine or other specific medication for their myotonia. The range show patients being mildly to severely affected, see Table 3.

**Table 3:** Characteristics of the DM1 group

Characteristics	Mean ± SD	Range
Disease duration years	18.9 ± 6.7	9.0–28.0
CTG kb	1.4 ± 0.8	0.3–3.1
TUG seconds	6,1 ± 1.6	3.9–10.0
6MWT meters	399.2 ± 114.3	140.0–615.0
FVC % *	74.2 ± 18.9	32–103
	Median	Range
RMI questionnaire	14.0	11.0–15.0

*; *n* = 18, FVC; forced vital capacity, 6MWT; 6-min walk test, TUG; timed up and go, RMI; Rivermead mobility index, kb; kilobyte

### Case-control comparison of fat infiltration measured by MRI

None of the MRI measures showed significant left/right differences. We therefore only report findings from the right side, see Additional file 1: Table S5. There was a significant difference between cases and controls in the degree of fat infiltration in all muscles measured except for the psoas and the transverse abdominal muscles (Table 4). For details about relations between muscle fat infiltration and BMI and age see Additional file 1: Table S3.

**Table 4:** Differences in trunk muscle fat infiltration between 20 DM1 patients and 20 controls

Fat infiltration	DM1 patients		Controls		Difference
Muscles	Median	Range	Median	Range	p-level
a: Differences in individual muscles. Adjusted *p* level = 0.005.					
Cranial rectus abdominis	2	0–5	1	0–2	< 0.001
Caudal rectus abdominis	2	0–5	1	0–2	0.001
External abdominal oblique	2	0–3	1	0–2	0.024
Internal abdominal oblique	1	0–3	1	0–2	0.011
Transvers abdominal	0	0–2	0	0–0	0.101
Psoas	0	0–1	0	0–1	0.161
Erector spinae above L2/L3	2	1–4	1	0–2	< 0.001
Erector spinae L2/3-L4/5	2	1–3	1	0–2	< 0.001
Erector spinae below L4/L5	2	1–5	1	0–2	< 0.001
Gluteus maximus	1	0–3	1	0–2	0.018
b: Differences in sum scores of fat infiltration					
Sum trunk flexors	9	(0–13)	4	(0–7)	0.001
Sum trunk extensors	6	(3–11)	3	(0–6)	0.000

The scores of muscle fat infiltration are according to the Mercuri-score

**Fig. 2:**
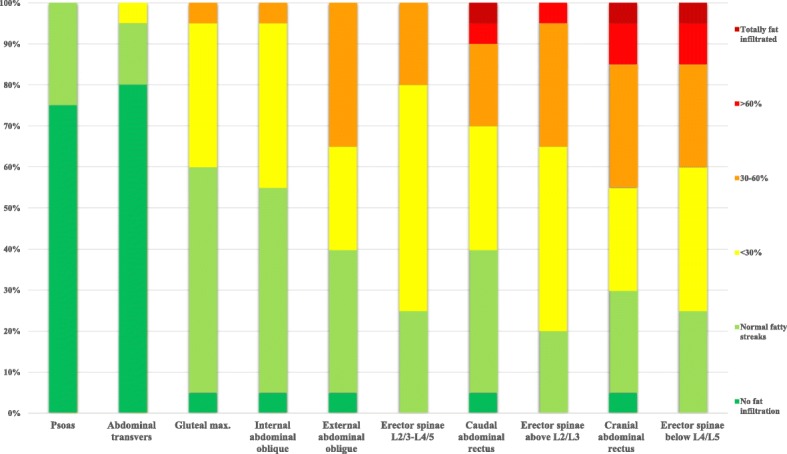
Distribution of fat infiltration in the different trunk muscles in the 20 DM1 patients. Muscles in the DM1 group are sorted from least to most severely fat infiltration. The fat infiltration categories 1 and 2 colored green in the fig. are regarded to be within the normal variation

**Fig. 3:**
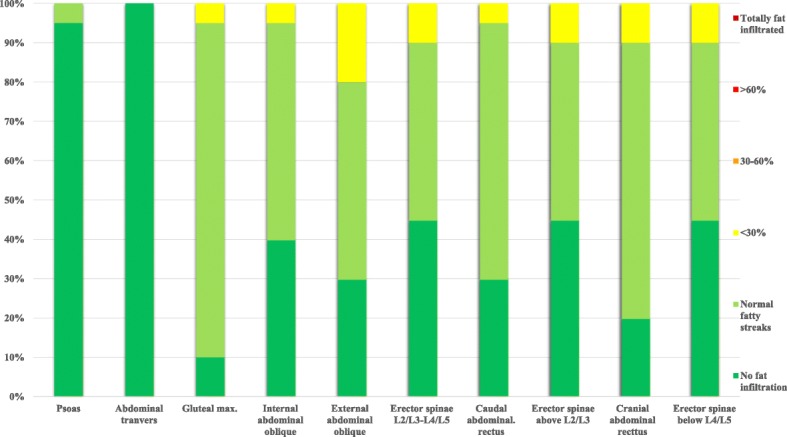
Distribution of fat infiltration in the different trunk muscles in the 20 controls. Muscles in the control group is sorted from left to right in the same order as for the DM1 group in Fig. 1. The fat infiltration categories 1 and 2 colored green in the fig. are regarded to be within the normal variation

**Fig. 4:**
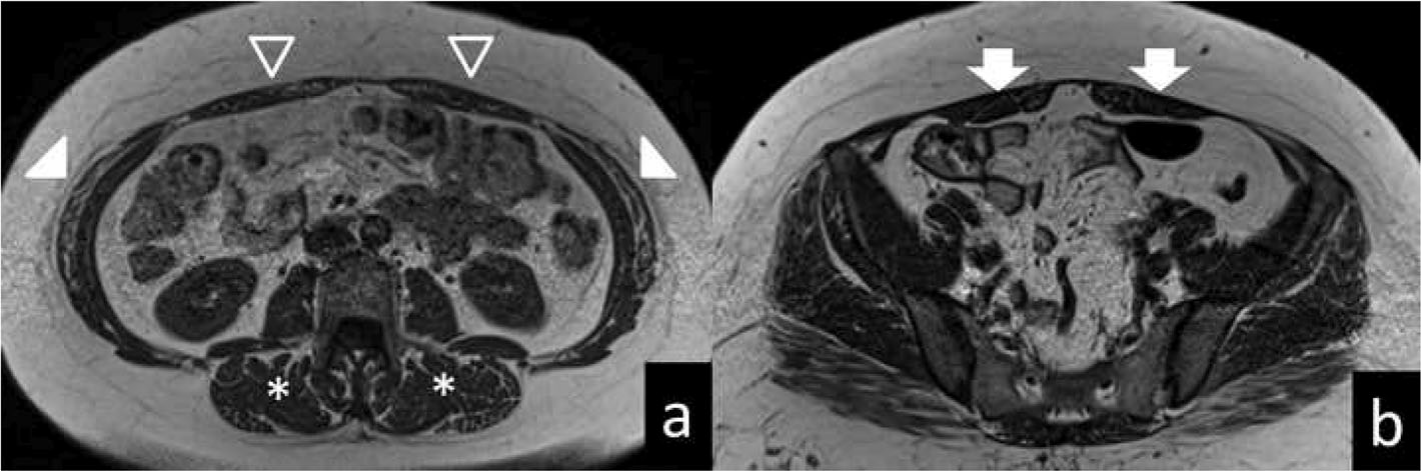
MRI of case. 40-year-old DM1 patient. Transversal T1-weighted sequence in L3/L4- (**a**) and S1/S2-level (**b**) show fat infiltration grade 3 in the cranial rectus abdominis (open arrow head) and the external oblique’ s (arrow head), and fat infiltration grade 2 in the caudal rectus abdominis (arrow) and in the erector spinae (*)

**Fig. 5:**
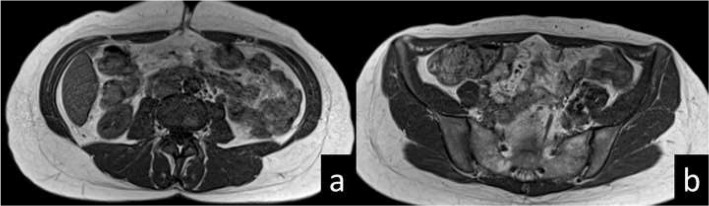
MRI of control. 40-year-old control. Transversal T1- weighted sequence in L3/L4- (**a**) and S1/S2-level (**b**) show normal muscles (score 0 and 1 on the Mercuri – score)

The degree of fat infiltration varied within the patient group. Some patients had total fat replacement in several muscles while others had normal findings. How the different fat infiltration categories are distributed in the various muscles in the DM1 and the control group is displayed in Figs. 2, 3, 4 and 5.

In the patient group the cranial rectus abdominis was the most severely affected muscle, with 45% of the 3 highest (> 30–100%) fat infiltration categories present. The second most affected muscle was the erector spinae below L4/L5 with 40% of the three highest categories present. For both the erector spinae and the rectus abdominis, the cranial part of the muscles was most frequently fat infiltrated, displaying a cranial to caudal pattern (Fig. 1). The abdominal transverse had the smallest amount of fat infiltration while the psoas was completely within the normal range in the patient group. In the control group, there were normal values and all had the lowest fat infiltration category (Figs. 2 and 3).

### Case-control comparison of muscle size measured by MRI

Muscle size was significantly different between the groups for three of the measured muscles: both levels of the rectus abdominis and the erector spinae L5/S1. The differences are large for all these muscles and most prominent for the cranial rectus abdominis. The findings in individual muscles and their group differences are presented in Table 5. For details about muscle size and gender in the two groups see Additional file 1: Table S4.

**Table 5:** Diameter and area of trunk muscles in 20 DM1patients and 20 controls

	DM1 patients		Controls		Difference		
**Muscles**	Mean ± SD	Range	Mean ± SD	Range	t	*p* level	Cohens - d
a: Differences in muscle size for individual muscles. Adjusted p level = 0.005							
Cranial rectus abdominis, mm	9 ± 2.5	4–14	12.3 ± 2.3	9–19	4.19	< 0.001	1.33
Caudal rectus abdominis, mm	10 ± 4.6	2–17	15.0 ± 3.7	10–27	3.55	0.001	1.12
External abdominal oblique, mm	8 ± 2.3	6–14	9.5 ± 2.9	5–15	1.28	0.208	0.35
Internal abdominal oblique, mm	8.3 ± 2.8	3–15	9.3 ± 2.0	6–13	1.09	0.281	0.41
Abdominal transverse, mm	3.6 ± 1.2	2–6	3.5 ± 1.1	2–6	0.14	0.893	0.04
Sum: oblique and transvers, mm	20.4 ± 4.7	14–32	22.3 ± 5.4	15–33	1.15	0.256	0.36
Psoas, mm^2^	1331.0 ± 372.5	752–2151	1436.7 ± 534.6	757–2605	0.73	0.473	0.23
Erector spinae L1/L2, mm^2^	1812.0 ± 433.7	989–2486	2106.0 ± 714.8	1242–3681	1.57	0.124	0.50
Erector spinae L3/L4, mm^2^	2069.3 ± 445.4	1471–3110	2391.2 ± 685.2	1252–4129	1.76	0.088	0.56
Erector spinae L5/S1, mm^2^	705.3 ± 351.7	0–1276	1010.1 ± 286.1	660–1576	3.00	0.005	0.95
b: Differences in muscle size for sum scores							
Muscle size	DM1patiens		Controls		Difference		
Muscles	Mean	Range	Mean	Range	p-level		Cohen’s *d*
Trunk flexors mm	40	(24–56)	50	(35–73)	0.002		1.05
Trunk extensors mm^2^	4587	(2727–6707)	5507	(3281–86,389)	0.030		0.71

Millimeter = mm, square millimeter = mm^2^. The table displays the mean score, standard deviation and range, effect sizes d, and t with its p level are reported for each muscle

### Edema

Edema in trunk muscles was found in nine patients and only one control. Further, edema was present in both trunk flexors and trunk extensors, although not present in the abdominal rectus below the umbilicus and the abdominal external oblique, nor in the erector spinae above L2/L3 or the psoas. Six patients had edema in the trunk flexors (abdominal rectus above the umbilicus: one patient, abdominal oblique internus: two patients, abdominal transverse: three patients), six patients had edema in the trunk extensors (erector spinae between L2/L3_L4/L5: 3 patients, and erector spinae below L4/L5: 3 patients) and one patient had edema in the gluteal maximus. Patients with edema had significant higher levels of fat infiltration in trunk extensors (*p* = 0.022), and significant lower muscle size in trunk extensors (*p* = 0.026).

### Correlation between, MRIs, CTG size and disease duration

The disease specific CTG-expansion size was not significantly correlated with any MRI measurements; nor sum scores of fat infiltration or muscle size in the trunk flexors and trunk extensors. Disease duration was related to fat infiltration in the trunk flexors (*rho* = 0.47, *p* = 0.037) and the gluteal maximus (*rho =* 0.57, *p =* 0.008).

### Correlation between MRI findings and MMT within the DM1 group

Fat infiltration and muscle size were both related to muscle strength. The sum score of fat infiltration in the abdominal flexors was related to trunk extension strength. On the other hand, the sum score of muscle size in the abdominal flexors was related to trunk flexion strength (Table 6).

**Table 6:** Correlations between MRI findings and MMT 0–3 in the DM1 patient group

MRI findings	Muscle strength	
Muscles	Trunk Extension	Trunk Flexion
Sum fat in trunk flexors	*rho*-0.523*, *p* = 0.018	*rho*-0.291, *p* = 0.214
Sum muscle size in trunk flexors	*r* = 0.219, *p* = 0.353	*r* = 0.474*, *p* = 0.035
Sum fat in trunk extensors	*rho*-0.427, *p* = 0.060	*rho*-0.177, *p* = 0.445
Sum muscle size in trunk extensors	*r* = 0.140, *p* = 0.556	*r* = 0.319, *p* = 0.171

*Spearman’s rho for correlations to muscle strength. Fat infiltration is scored according to the Mercuri-score, muscle size is measured in millimeters for trunk flexors and millimeters^2^ for trunk extensors

**Table 7:** Correlations between MRI findings and function

MRI measures	Respiratory function	Motor function		
Muscles	FVC n18	TUG	RMI	6MWT
Sum fat in trunk flexors	−0.487* p = 0.040	0.630** p = 0.003	−0.628**p = 0.003	−0.404p = 0.077
Sum muscle size in trunk flexors	0.551*p = 0.018	−0.259p = 0.270	0.447*p = 0.048	0.377p = 0.102
Sum fat in trunk extensors	−0.220 *p* = 0.380	0.305 *p* = 0.191	−0.407 *p* = 0.075	−0.12 *p* = 0.960
Sum muscle size in trunk extensors	0.349 *p* = 0.156	−0.004p = 0.987	0.401p = 0.079	0.124p = 0.604

*Spearman’s rho for fat infiltration, and Pearson’s r for muscle size. Fat infiltration is scored according to the Mercuri-score, muscle size is measured in millimeters for trunk flexors and millimeters^2^ for trunk extensors

### Correlation between MRI findings and motor and lung function in the DM1 group

Fat infiltration in the trunk flexors were strongly correlated with the RMI and TUG, but not to the 6 MWT. Both fat infiltration and muscle size in the trunk flexors was significantly correlated to FVC, the strongest correlation was found to muscle size (Table 7). FVC was also correlated to the CTG expansion size (*r =* − 0.67, *p =* 0.003). A regression model with muscle size of the cranial rectus abdominis and CTG size as independent variables, and FVC as dependent variable, was calculated, and R square for this model was 0.72, both covariates muscle size (β = 0.562, *p =* 0.001) and CTG (β = 0.537, *p =* 0.001) had independent contributions.

## Discussion

To our knowledge, this is the first study of fat infiltration and muscle size in trunk muscles in DM1 patients comparing cases and healthy controls. It is also the first study of trunk muscle involvement measured by MRI in relation to results from clinical testing of strength and other muscle function tests as well as respiratory variables. We found a statistically significant difference in fat infiltration and muscle size between patients and age-matched healthy controls. In addition, we have shown that there are strong relations between MRI findings in the patient group and impairment of both motor performance and respiratory function.

### Differences in fat infiltration and muscle size

Fat infiltration was significantly different between cases and controls for all the measured muscles except for psoas, which seems spared by DM1, a result in line with Park et al. [16]. Our clinical findings are in line with our previous study on a larger group of patients [7], where we found impaired trunk muscle strength and that this was significantly related to mobility and balance. However, in this previous study we did not include MRIs. Our findings of fat infiltration in trunk extensors and trunk flexors in DM1 are also in line with other MRI studies of both trunk muscles and extremity muscles in DM1, and other myopathies [13, 14, 16]. However, we included measures of muscle size and levels of muscles in DM1, not previously investigated by MRI; the middle and cranial parts of the lumbar erector spinae and a cranial and caudal part of the rectus abdominis. The severity of fat infiltration found in the trunk muscles, are in line with fat infiltration found in extremity muscles and thus most likely indicates DM1 myopathy [16, 37]. This high degree of fat infiltration in trunk muscles is also found in other muscular dystrophies [6, 13]. The fact that only three muscles showed case control differences in muscle size compared to the case control differences in most muscles when it comes to fat infiltration may indicate a continuum: A certain amount of fat infiltration must be present before muscle size decreases.

### Edema

The finding of edema in trunk muscles in nine patients and only one control indicates edema to be part of myopathic changes in trunk muscles in DM1, which is in line with previous findings investigating edema in trunk extensors in this patient group [14]. The fact that edema is found in both trunk flexors and trunk extensors is in line with our experience [7], as well as other studies finding both muscle groups being affected in DM1 patients [13, 16]. Patients with edema had significantly more fat infiltration and atrophy in the trunk extensors, than patients without edema. This may implicate that the processes occurs simultaneous, but still are in line with other studies suggesting edema to be previous to fat infiltration [14].

### MRI findings and respiratory and motor function

We find a high and significant correlation between the sum score of muscle size in the trunk flexors and FVC. We think that this finding reflects the importance of the rectus abdominis in forceful expiration [12]. There was also a significant correlation between the sum score of fat infiltration in the trunk flexors and FVC, which is contrary to a previous study [16]. However, muscle size was the most correlated to FVC, and this finding is in line with muscle volume as the most predictive value of strength function [17]. Trunk muscles in DM1 may clearly be fat infiltrated and atrophied, both myopathic changes related to muscle strength and FVC, and should not be neglected in this patient group. Rather, since respiratory function has impact on life expectancy, we suggest that trunk muscle strength should be thoroughly assessed in the clinical follow up of these patients.

Fat infiltration in the trunk flexors is correlated to performance on TUG. Stabilization, flexion and rotation of the trunk are involved in TUG and therefore all abdominal muscles are involved in this task. These findings are of importance in the understanding of how balance may be influenced by trunk impairments in the DM1 group and support our previous finding of a relation between strength in trunk muscles and TUG [7]. Bachasson et al. found that the postural stability and gait in DM1 patients was disturbed and related to strength in the distal part of the lower extremities. The authors also argue that changes in pelvic tilt may play a role in gait disturbances in the DM1 group. However, this study did not investigate the trunk muscles [38] . Our findings suggest trunk muscles should be included when postural stability or balance is investigated in the DM1 group. In patients with facioscapulohumoral muscular dystrophy (FSHD) gait function has been shown to be more related to fat infiltration in the trunk muscles than to fat infiltration in the lower extremities [39]. Interestingly, RMI, the measure of general mobility, was significantly related to both fat and size in the abdominal and the back muscles in our patients. This finding is understandable as this test is composed of gross motor movements, some that are dependent on all trunk muscles working against gravity, which demands strength levels above grade 2 (muscle strength score indicating ability to move the body part against gravity). Since RMI involve all trunk muscles, it is possibly more sensitive to change and seems to be able to predict myopathy in trunk muscles. However, which strategy a subject uses, when performing the different tasks of RMI is not fixed and allows for compensation such as using the arms to get from a supine to a sitting position. An accurate observation of how the tasks in the RMI are performed by DM1 patients would be of interest, since this may indicate trunk function impairment.

The 6MWT performed by our patients was not significantly related to any of the MRI findings. One explanation may be the low strength needed from the trunk muscles in walking [40]. However, an MRI study that investigated the thoracic levels of the erector spinae in DM1 patients, identified significant relations between these muscles and the 6MWT [16]. This might indicate that the more cranial parts of the paravertebral muscles are the most important trunk muscles for walking.

### MRI findings and relation to trunk muscle strength

The correlation between fat infiltration in the trunk flexors and trunk extension may be explained by the opposing role the trunk flexors have to trunk extensors and the need for co-contraction of antagonists in stabilizing the spine [41, 42]. A lack of correlation between trunk extension strength and fat infiltration in the trunk extensors, may be due to the higher frequency of fat infiltration present in the cranial parts, which were not included in this study, and to a greater demand of power from this part of the erector spinae in the trunk extension test performed [43]. The only significant correlation of muscle size and strength was between the trunk flexors and trunk flexion strength, measured by the curl up, a result in line with muscle volume as the most predictive value of strength function [17]. Compensation from other muscles may be the reason for the lack of relations between muscle size and trunk extension. Only the lumbar part of the erector spinae was included in this MRI study, and both the thoracic parts of the erector spinae as well as cervical extensors and other trunk extensors are known to contribute in trunk extension [44].

### MRI and CTG expansion size

CTG expansion size was not significantly correlated with any MRI measure; nor fat infiltration or muscle size, in neither trunk flexors or trunk extensors. This result may be due to the mosaic expression of CTG repeats in different tissues in DM1 patients; a higher number of CTG repeats are found in skeletal muscles compared to blood [45]. A significant correlation between MRI measured fat infiltration in trunk flexors and CTG repeats measured in blood is documented by Park. However, in the same study a correlation between fat infiltration in trunk extensors was not significant [16]. This finding may be taken into account when standards of care are recommended. Health professionals should be aware that decreased respiratory function probably might develop early and independent of CTG size.

### Strengths and limitations

This study of well characterised and matched case-control groups was sufficiently powered for the main question of differences in findings between cases and controls. However, it might be that the question of muscle size would have profited on a larger sample. Correlations between MRI derived measures and disease specific measures in the DM1 group are underpowered (*n* = 20). DM1 patients with pacemakers were excluded, and our results might therefore not be generalized to the whole group of DM1 patients. On the other hand, the correlations we have identified may be stronger in a group where symptoms could be more severe, such as patients with pacemakers [46]. A strength of the present study is our optimized MRI protocol, reaching acceptable examination time and especially breath-holding times for this patient group. T1 Dixon sequences may have given us more exact qualitative data [47, 48], but would have prolonged the examination time for the patients. We therefore found it not suitable for the present study and patient population. Another strength of this study is the two experienced radiologists analyzing the MRIs and reaching a mean ICC score of 0.90.

## Conclusion

The presence of fat infiltration and atrophy in trunk muscles in patients with DM1 shows that these muscles are affected by DM1 myopathy. Fat infiltration was correlated with reduced balance, and both fat infiltration and increased atrophy was correlated with reduced respiratory function. These findings are of importance for clinical management of the disease and could be useful as an additional outcome measure in future intervention studies.

## Additional file

Additional file 1: **Table S1.** Characteristics of the DM-group: muscle strength (Adapted MMT 0–3) in extremity muscle groups and neck flexion. **Table S2.** Grading of muscle strength in trunk according to Medical Research Council (MRC) 0–5 scale for MMT. **Table S3.** Correlation between the MRI measures and age and BMI in patients and controls. **Table S4.** Group differences for muscle size between genders in the 20 DM1 patients and 20 controls. **Table S5.** Differences between left and wright side in individual muscles. (DOCX 20 kb)

## Data Availability

The datasets generated and/or analysed during the current study are not publicly available due to the consent form used, some limitation of data sharing may apply, but are available from the corresponding author on reasonable request.

## Abbreviations

6MWT: Six-minute walk test
BiPAP: Bilevel positive airway pressure
BMI: Body mass index
CTG: Cytosine thymine guanine trinucleotide repeat
DM1: Myotonic dystrophy type 1
DMPK: Myotonic dystrophy protein kinase gene
FOV: Field of view
FVC: Forced vital capacity
MMT: Manual muscle strength tests
MRC: Medical research council
MRI: Magnetic resonance imaging
RMI: Rivermead mobility index
SPSS: Statistical package for the social sciences
TUG: Timed up & go
