# Supplementary material for: MRI of trunk muscles and motor and respiratory function in patients with myotonic dystrophy type 1
Source: BMC Neurol. 2019 Jun 19;19:135. doi: 10.1186/s12883-019-1357-8 (PMC6582475; doi:10.1186/s12883-019-1357-8)
Supplement: Supplementary file 1 — Table S1. Characteristics of the DM-group: muscle strength (Adapted MMT 0–3) in extremity muscle groups and neck flexion. Table S2. Grading of muscle strength in trunk according to Medical Research Council (MRC) 0–5 scale for MMT. Table S3. Correlation between the MRI measures and age and BMI in patients and controls. Table S4. Group differences for muscle size between genders in the 20 DM1 patients and 20 controls. Table S5. Differences between left and wright side in individual muscles. (DOCX 20 kb) [file 12883_2019_1357_MOESM1_ESM.docx]

**Additional file 1**

**Table S1** **Characteristics of the DM-group: muscle strength (Adapted MMT 0-3) in extremity muscle groups and neck flexion.**

| **Muscles** | **Median** | **Min** | **Max** |
| --- | --- | --- | --- |
| Shoulder abduction | 2 | 2 | 3 |
| Elbow flexion | 2 | 2 | 3 |
| Elbow extension | 2 | 2 | 3 |
| Hip flexion | 3 | 2 | 3 |
| Hip Extension | 3 | 3 | 3 |
| Knee extension | 3 | 3 | 3 |
| Knee flexion | 3 | 2 | 3 |
| Ancle dorsalflexion | 2 | 1 | 3 |
| Ancle plantarfleks | 2 | 1 | 3 |
| Wrist extension | 2 | 2 | 3 |
| Neck flexion | 1 | 1 | 3 |

**Table S2** **Grading of muscle strength in trunk according to Medical Research Council (MRC) 0-5 scale for MMT**

| **Muscles** | **Test position** | **Fixation** | **Movement** | **Resistance** | **Grades** |
| --- | --- | --- | --- | --- | --- |
| Trunk flexors | Supine, legs straight | At feet after trunk curl | Full trunk curl, then sit up | Arms behind neck | **5** |
|  |  |  |  | Arms folded over chest | **4** |
|  |  |  |  | Arms along the body | **3** |
|  |  | None | Posterior tilt pelvic | Arms along the body | **2** |
| Back extensors | Prone | On tights or hips | Full extension of the spine | Behind neck | **5** |
|  |  |  |  | Arms along the body, and by examiner | **4** |
|  |  |  |  | Arms along the body | **3** |
|  |  |  | Extension of the spine | Arms along the body | **2** |
|  |  |  |  |  |  |

**Table S3** **Correlation between the MRI measures and age and BMI in patients and controls.**

| **Muscle fat infiltration and age:** | **DM1 patients** | **Controls** |
| --- | --- | --- |
| Sum fat trunk extensors | rho=0.632 p=0.003 | rho=0.585 p=0.007 |
| Sum fat trunk flexors | rho=0.444 p=0.050 | rho=0.500 p=0.025 |
| **Muscle fat infiltration and BMI:** |  |  |
| Sum fat trunk flexors | n.s | rho=0.488 p=0.034 |
| Gluteal Max | rho=0.574 p=0.008 | rho=0.527 p=0.020 |
| **Muscle size and age:** | n.s | n.s |
| **Muscle size and BMI:** |  |  |
| Sum size trunk extensors | n.s | r=0.571 p= 0.011 |
| Psoas | n.s. | r=529 p=0.020 |

n.s= not significant correlations. * p=<0.05, **p=<0.01

There are several muscles sig. correlated to age and BMI in the control group than in the DM1 group.

**Table S4** **Group differences for muscle size between genders in the 20 DM1 patients and 20 controls.**

| **DM1 group** | **Women** | **Men** | **Difference** |  |  |
| --- | --- | --- | --- | --- | --- |
| **Muscle size** | **mean** ± **SD** | **Mean** ± **SD** | t | p | d |
| Sum trunk extensors mm^2^ | 4207 ± 1032 | 5157± 779 | 2.35 | 0.031 | 1.04 |
| Sum trunk flexors mm | 36 ± 7 | 46 ±7 | 3.04 | 0.007 | 1.4 |

| **Control group** | **Women** | **Men** | **Difference** |  |  |
| --- | --- | --- | --- | --- | --- |
| **Muscle size mm** | **mean** ± **SD** | **Mean** ± **SD** | t | p | d |
| Sum trunk extensors mm^2^ | 4545 ± 617 | 6950 ±1352 | 4.7 | 0.001 | 2.3 |
| Sum trunk flexors mm | 45 ± 7 | 55 ± 11 | 2.3 | 0.034 | 1.08 |

Gender seems to be influencing muscles size different in the DM1 and control group;

In the control group the difference is larger for trunk extensors than trunk flexors. In the DM1 group the difference is larger for on trunk flexors than trunk extensors. The groups are small men n 8, women n 12.

**Table S5 Differences between left and wright side in individual muscles**

| **Muscle size**  **(mm and mm^2)^** | t | p | **Muscle fat infiltration (Mercurie score)** | p |
| --- | --- | --- | --- | --- |
| Cranial rectus abdominis, mm | 0.28 | 0.781 | Cranial rectus abdominis | 1 |
| Caudal rectus abdominis, mm | 0.67 | 0.504 | Caudal rectus abdominis | 0.16 |
| External abdominal oblique, mm | 0.30 | 0.766 | External abdominal oblique | 1 |
| Internal abdominal oblique, mm | 0.69 | 0.493 | Internal abdominal oblique | 0.56 |
| Abdominal transverse, mm | 0.43 | 0.667 | Abdominal transverse | 0.32 |
| Psoas, mm^2^ | 0.37 | 0.707 | Psoas | 1 |
| Erector spinae L1/L2, mm^2^ | 1.75 | 0.087 | Erector spinae above L2/L3 | 1 |
| Erector spinae L3/L4, mm^2^ | 1.37 | 0.177 | Erector spinae L2/ L3_L4/L5 | 1 |
| Erector spinae L5/S1, mm^2^ | 1.54 | 0.132 | Erector spinae below L4/L5 | 1 |
|  |  |  | Gluteus maximus | 0.55 |

Paired sample t-tests were used for comparison of left vs right side muscle size; Wilcoxon signed-rank test were used for comparison of left vs right side degree of fat infiltration. Adjusted p level = 0.005

There are no significant left wright differences in any muscles nor for fat infiltration or muscle size.
